# Effect of Temperature on the Growth Dynamics and Cell Viability of *Chlorella vulgaris* Under Elevated CO_2_ Conditions

**DOI:** 10.3390/plants15213364

**Published:** 2026-10-30

**Authors:** Vadim A. Pavlov, Marina E. Vavilkina, Maksim A. Kravets, Elizaveta M. Kovalenko, Anatoly V. Grigorenko, Mikhail S. Vlaskin

**Affiliations:** Joint Institute for High Temperatures of the Russian Academy of Sciences, 125412 Moscow, Russia; vavilkina-marina@rambler.ru (M.E.V.); kravets.ma@phystech.edu (M.A.K.); lizloom@mail.ru (E.M.K.); presley1@mail.ru (A.V.G.)

**Keywords:** *Chlorella vulgaris*, temperature, photobioreactor, carbon dioxide, microalgal growth, specific growth rate, cell viability

## Abstract

*Chlorella vulgaris* is a promising platform for biomass production and CO_2_ fixation, while temperature strongly affects cultivation performance. Here, the effect of temperature on growth and cell viability was investigated in 10 L column bubble-type photobioreactors under an elevated, intermittently restored CO_2_ atmosphere. Cultures were grown for 18 days at nominal temperatures of 19, 23, 27, and 31 °C using two independent photobioreactors per temperature. Continuous monitoring confirmed mean culture temperatures of 19.12 ± 0.42, 23.09 ± 0.09, 27.19 ± 0.20, and 30.96 ± 0.44 °C, respectively. Using an OD_750_-to-biomass coefficient previously established in our laboratory for this *C. vulgaris* culture by paired OD_750_ and gravimetric dry-weight measurements (0.4 g L^−1^ per OD unit), final OD_750_-derived biomass estimates were 5.43, 5.82, 5.90, and 4.05 g L^−1^ at 19, 23, 27, and 31 °C, respectively, and corresponding average OD-derived productivities were 0.282, 0.303, 0.308, and 0.205 g L^−1^ d^−1^. Reactor-level analysis detected no significant productivity differences among 19, 23, and 27 °C, whereas the OD-derived productivity at 31 °C was significantly lower. Non-viable cells appeared earlier at 31 °C; at day 18, the group-level viable-cell fractions were 93.3% at 19 °C and 91.7% at 31 °C. Because the OD_750_ conversion coefficient was not independently validated at each temperature and the day-18 count-derived cell densities at 19 and 31 °C were similar, the magnitude of the apparent biomass difference should be interpreted as an OD_750_-derived response rather than as a validated dry-biomass difference. The data therefore support a temperature-dependent cultivation response at 31 °C but do not resolve its physiological or gravimetric basis.

## 1. Introduction

Microalgae have attracted considerable interest as a biotechnology platform for biomass, biofuels, feed and food ingredients, pigments, and other bioactive compounds, as well as for wastewater treatment and biological carbon dioxide fixation [1,2,3]. Among microalgae, *Chlorella vulgaris* is one of the most widely studied species because of its rapid growth, substantial biomass accumulation, and suitability for cultivation in different technological systems [1,2]. Closed photobioreactors are widely used for controlled microalgal cultivation because they allow regulation of light, temperature, gas exchange, and hydrodynamic conditions, thereby reducing the effects of uncontrolled environmental factors [4,5].

Microalgal growth in photobioreactors is determined by interacting parameters including light intensity and distribution, temperature, mixing and aeration, inorganic carbon availability, nutrient-medium composition, and the initial physiological state of the culture [4,5,6]. Because these factors interact, changing a single operating parameter can alter not only the final biomass concentration but also the entire temporal growth profile. In our previous studies, aeration intensity strongly affected biomass accumulation by *C. vulgaris* in laboratory photobioreactors [7], whereas the initial biomass concentration markedly altered the specific growth rate and cultivation kinetics [8]. Thus, the kinetics of biomass accumulation should be considered in addition to the final biomass yield.

Temperature is one of the principal factors controlling microalgal growth kinetics. It affects enzymatic reactions, photosynthetic activity, respiration, and other cellular processes, and the growth response is therefore typically nonlinear [9,10,11,12,13]. The approach proposed by Bernard and Rémond describes microalgal temperature responses using minimum, optimum, and maximum growth temperatures and accounts for the decline in productivity outside the favorable range [11]. Experimental studies of *C. vulgaris* likewise show that the temperature range supporting the most intensive growth depends on the strain, medium composition, illumination, and cultivation configuration and therefore cannot be treated as a universal characteristic of the species [9,10,12,13,14].

Temperature also affects the physiological state of the culture. Manhaeghe et al. showed that temperature and illumination alter the balance between photosynthetic growth and respiratory losses in *C. vulgaris* and that thermal stress may increase respiration [13]. Serra-Maia et al. analyzed the temperature response of *C. vulgaris* together with cell mortality and demonstrated that temperature affects both growth and population viability [12]. Consequently, reduced biomass accumulation at an unfavorable temperature may reflect not only greater cell death but also a lower division rate or reduced metabolic activity of the viable fraction of the culture.

The temperature response must also be considered in the context of inorganic carbon availability. During photoautotrophic cultivation, CO_2_ is the principal external carbon source, while its transfer from the gas phase to the culture medium depends on gas composition, gas-exchange intensity, and photobioreactor hydrodynamics [4,5,15,16,17,18]. Studies of *C. vulgaris* in laboratory and column photobioreactors show that biomass productivity depends on both temperature and CO_2_-supply conditions [10,15], while acclimation to changes in CO_2_ availability can itself be temperature dependent [16]. In an earlier study conducted by our group in the same 12 m^3^ gas-chamber system, *C. vulgaris* and other microalgae were cultivated at gas-phase CO_2_ concentrations ranging from atmospheric levels to 9 vol.%, providing direct methodological context for the elevated-CO_2_ regime used here [19].

Mixing, irradiance distribution, and CO_2_ mass transfer provide additional coupling between environmental conditions and biomass accumulation [17,18]. Consequently, temperature effects observed in a specific photobioreactor should be interpreted within the corresponding gas-supply and light regime.

The interaction between temperature and CO_2_ remains relevant in recent studies. Johnson examined combinations of temperatures from 25 to 32.5 °C and CO_2_ concentrations from atmospheric levels to 15%, with Chlorella productivity varying substantially among the factor combinations [20]. Thomas and Razaviarani developed a multi-parameter kinetic model for *C. vulgaris* that simultaneously considered temperature, light, CO_2_, O_2_, nitrogen, and phosphorus; their sensitivity analysis further emphasized the importance of temperature and carbon availability in determining biomass productivity [21]. However, results from such multi-factor models and small-scale experiments require validation for specific photobioreactor geometries and cultivation regimes.

Despite the large number of studies addressing temperature and CO_2_ effects on *C. vulgaris*, temperature responses are often characterized using final biomass concentration, average productivity, or a maximum specific growth rate [9,10,11,14,20]. These metrics do not necessarily show when differences between temperature treatments begin to emerge, while biomass concentration alone cannot distinguish changes in net biomass formation from changes in cell viability. Time-resolved studies combining biomass accumulation and viability remain comparatively limited [12,13].

Accordingly, simultaneous analysis of biomass concentration, growth dynamics, and cell viability at several temperatures within one photobioreactor system can provide a more complete cultivation-level characterization while avoiding the assumption of a universal temperature optimum.

The aim of this study was to investigate the 18-day temperature response of *Chlorella vulgaris* cultivated in column photobioreactors under an elevated-CO_2_ atmosphere. Cultures were maintained at nominal temperatures of 19, 23, 27, and 31 °C using two independent photobioreactors per treatment. Biomass concentration, interval growth metrics, culture-medium pH, cell viability, chamber CO_2_ dynamics, and titration-derived carbonate-system alkalinity were analyzed. The emphasis was placed on time-resolved cultivation performance rather than on identification of a universal optimum temperature or a specific physiological mechanism.

## 2. Results

### 2.1. Temperature Effects on OD_750_-Derived Biomass Estimates

**Figure 1:**
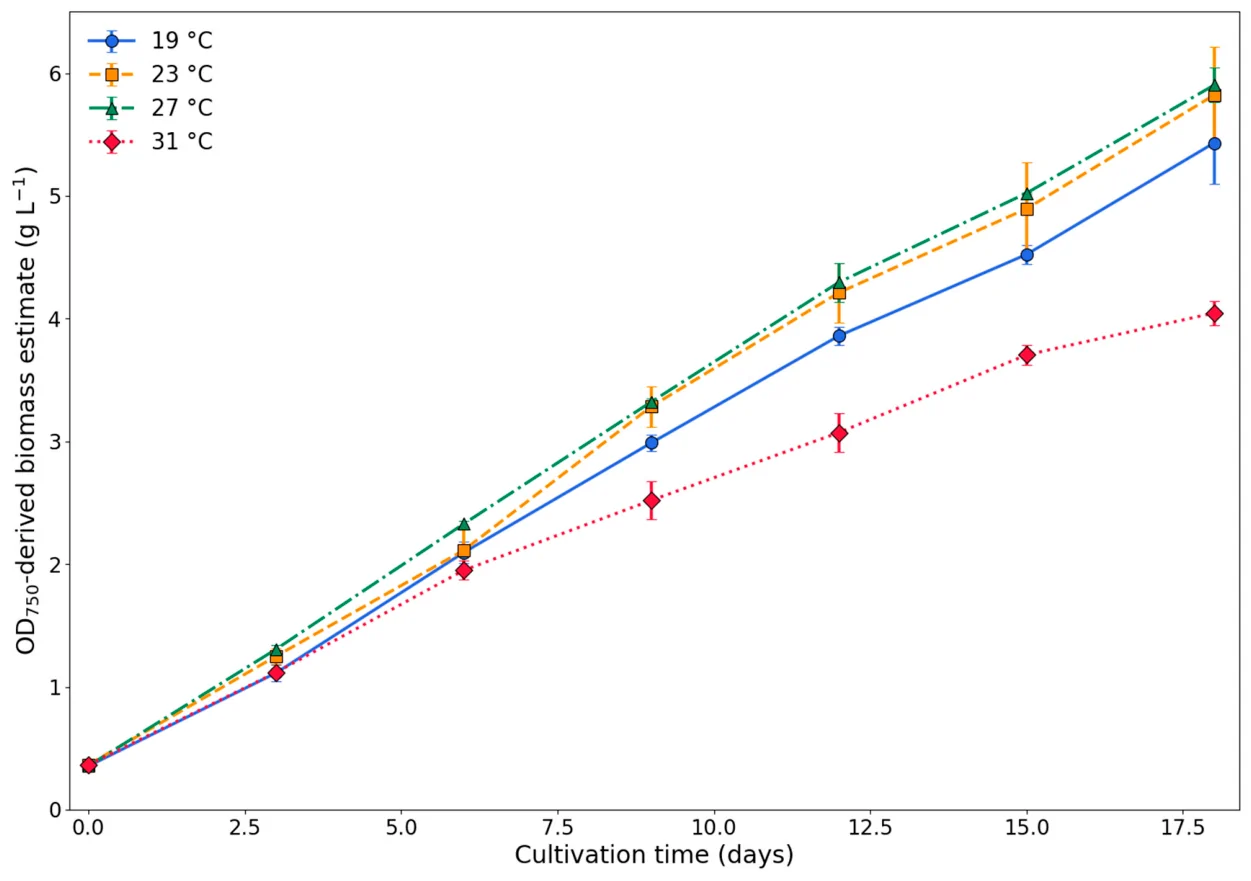
OD_750_-derived biomass estimates of *Chlorella vulgaris* during cultivation at 19, 23, 27, and 31 °C. Values were calculated using the common empirical coefficient of 0.4 g L^−1^ per OD unit and are presented as means of two independent photobioreactors; error bars show the standard deviation.

The OD_750_-derived biomass estimate increased throughout the 18-day cultivation period under all temperature regimes (Figure 1), although the temporal pattern depended on temperature.

At the beginning of the experiment, mean OD_750_-derived biomass estimates were 0.355, 0.367, 0.357, and 0.365 g L^−1^ at 19, 23, 27, and 31 °C, respectively, indicating comparable initial optical-density-derived conditions. During the first three days, the corresponding estimates reached 1.114, 1.251, 1.307, and 1.118 g L^−1^, respectively. Given the limited replication, these early differences are described without inferential ranking.

As cultivation proceeded, the OD_750_-derived trajectories at 19, 23, and 27 °C remained relatively close, whereas a clear reduction in the OD_750_-derived accumulation trajectory developed at 31 °C from the intermediate stages onward.

On day 18, mean OD_750_-derived biomass estimates were 5.43, 5.82, 5.90, and 4.05 g L^−1^ at 19, 23, 27, and 31 °C, respectively. Under the single empirical conversion coefficient used here, the 31 °C estimate was approximately 31% lower than the 27 °C estimate. Because the coefficient was not validated independently at each cultivation temperature, this percentage describes the difference in the OD_750_-derived biomass metric and should not be interpreted as a validated gravimetric dry-biomass difference. With two independent photobioreactors per temperature, the smaller differences among 19, 23, and 27 °C are interpreted descriptively rather than as statistically resolved differences.

**Figure 2:**
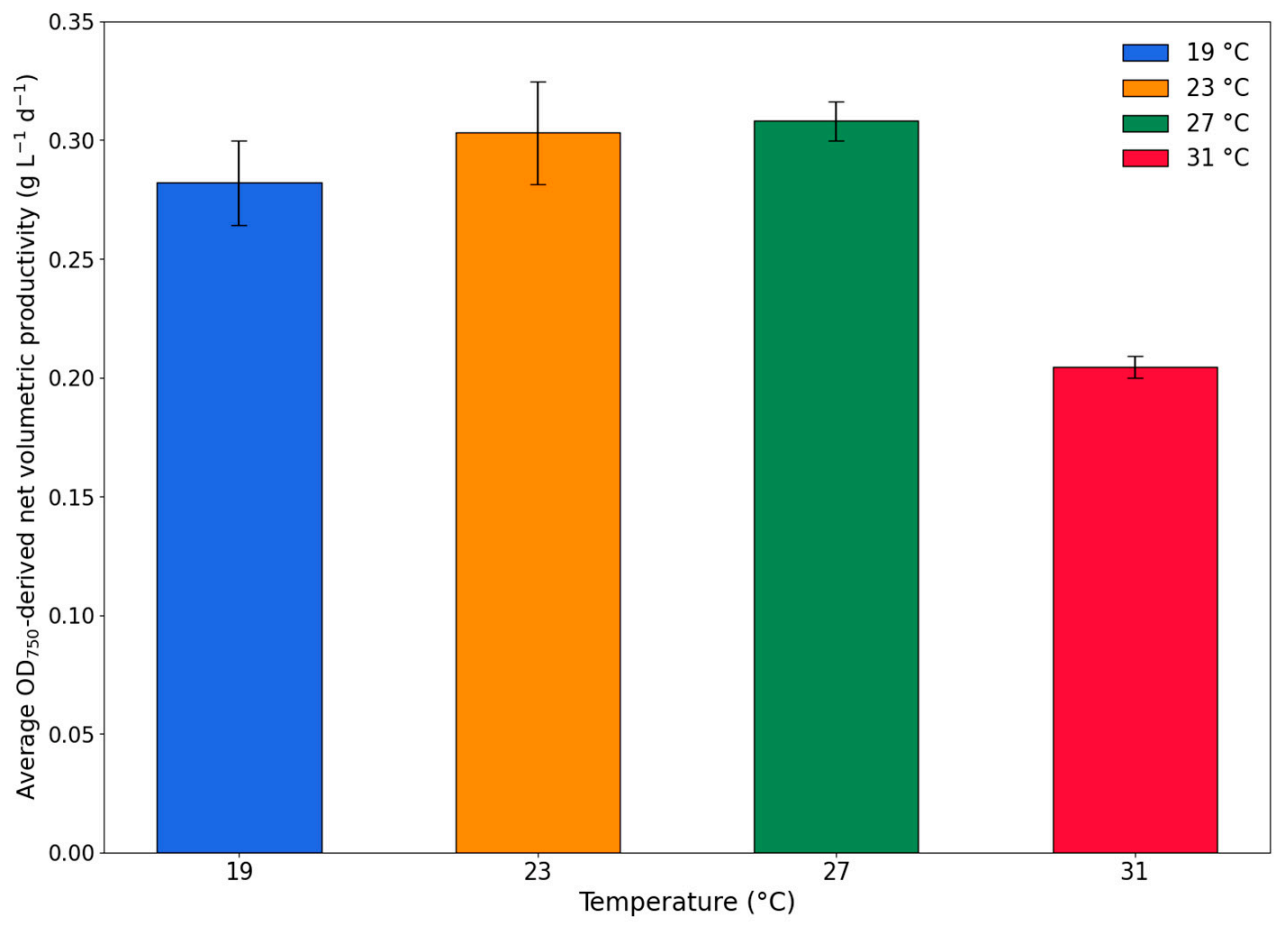
Average OD_750_-derived net volumetric productivity of *Chlorella vulgaris* as a function of cultivation temperature. Bars show the means of two independent photobioreactors; error bars show the standard deviation.

Average OD_750_-derived net volumetric productivity over the full 18-day cultivation period was 0.282, 0.303, 0.308, and 0.205 g L^−1^ d^−1^ at 19, 23, 27, and 31 °C, respectively (Figure 2). One-way ANOVA of the reactor-level OD-derived productivity indicated a significant temperature effect (F_3,4_ = 20.85, *p* = 0.0066). Tukey HSD comparisons showed no significant differences among 19, 23, and 27 °C (all adjusted *p* > 0.40), whereas the OD-derived productivity at 31 °C was lower than at 19 °C (*p* = 0.0217), 23 °C (*p* = 0.0092), and 27 °C (*p* = 0.0077). Because the absolute productivity values scale with the unverified OD_750_ conversion coefficient and only two independent PBRs were available per treatment, these inferential results are interpreted specifically as differences in the OD-derived metric.

### 2.2. Temporal Dynamics of Growth Rate and OD_750_-Derived Productivity

**Figure 3:**
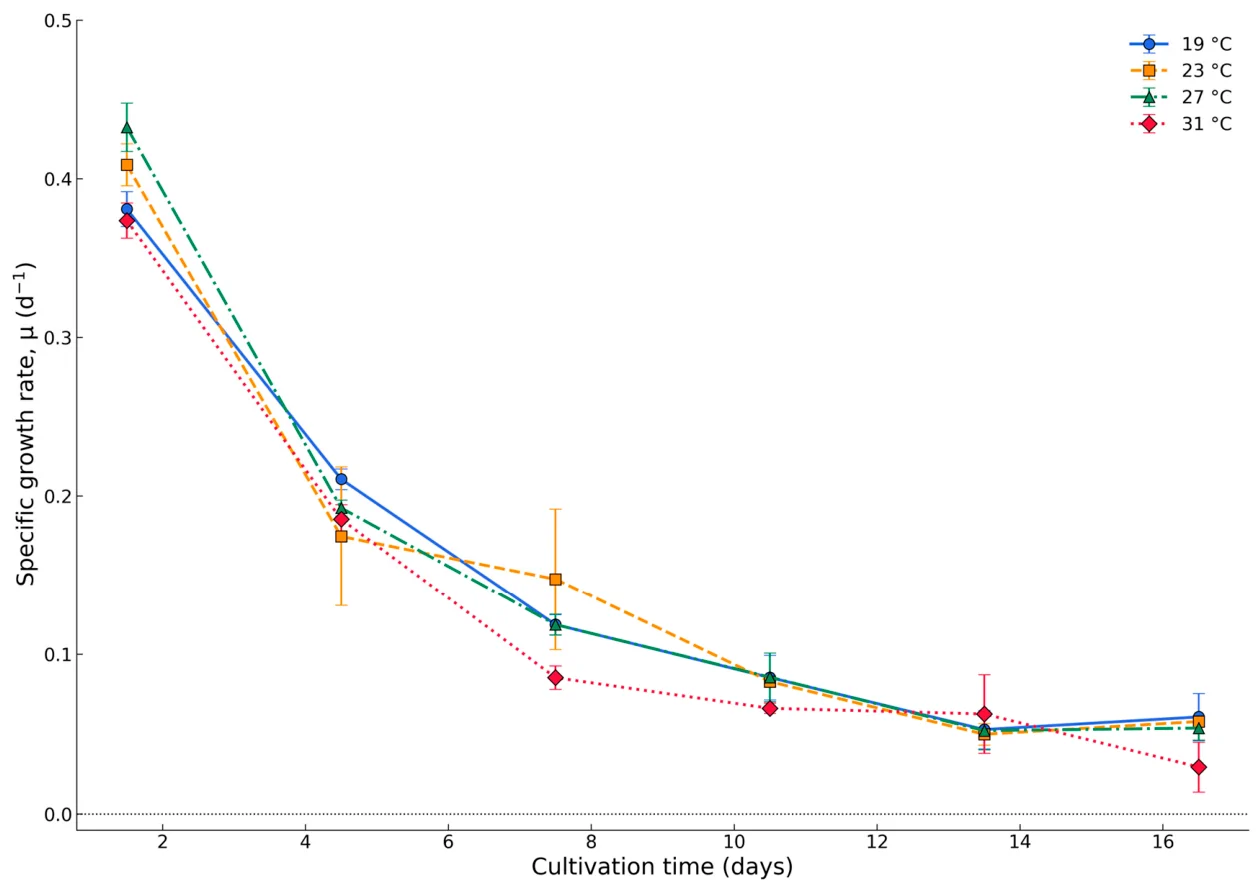
Interval-specific growth rate of *Chlorella vulgaris* during cultivation at different temperatures. Values are means of two independent photobioreactors; error bars show the actual standard deviation of the two reactor-specific values.

The calculated specific growth rate, obtained from ratios of successive OD_750_-derived estimates, was highest during the initial cultivation interval and progressively decreased under all temperature regimes (Figure 3). Because the same multiplicative OD-to-biomass coefficient appears in both terms of the logarithmic ratio, it cancels from the specific-growth-rate calculation.

During the first three days, interval-specific growth rates were 0.381, 0.409, 0.433, and 0.374 d^−1^ at 19, 23, 27, and 31 °C, respectively. Because the treatment means were based on two reactors, these values are used descriptively rather than to rank the 19–27 °C treatments.

The specific growth rate subsequently decreased under all temperature regimes. This decline partly reflects the logarithmic definition of specific growth rate as the OD-derived estimate increases. To describe the absolute change in the OD-derived metric more directly, interval volumetric productivity was also calculated. At 31 °C, mean OD-derived interval productivity decreased from 0.251–0.277 g L^−1^ d^−1^ during days 0–6 to 0.190 and 0.184 g L^−1^ d^−1^ during days 6–9 and 9–12, and to 0.114 g L^−1^ d^−1^ during days 15–18. Over the same final interval, values at 19, 23, and 27 °C were 0.303, 0.309, and 0.293 g L^−1^ d^−1^, respectively. These absolute productivity values inherit the uncertainty of the OD_750_-to-biomass conversion coefficient.

Thus, the lower OD_750_-derived estimate at 31 °C developed progressively during cultivation and was evident in both the specific-growth-rate profile and the OD-derived interval productivity.

### 2.3. Changes in Cell Viability

**Figure 4:**
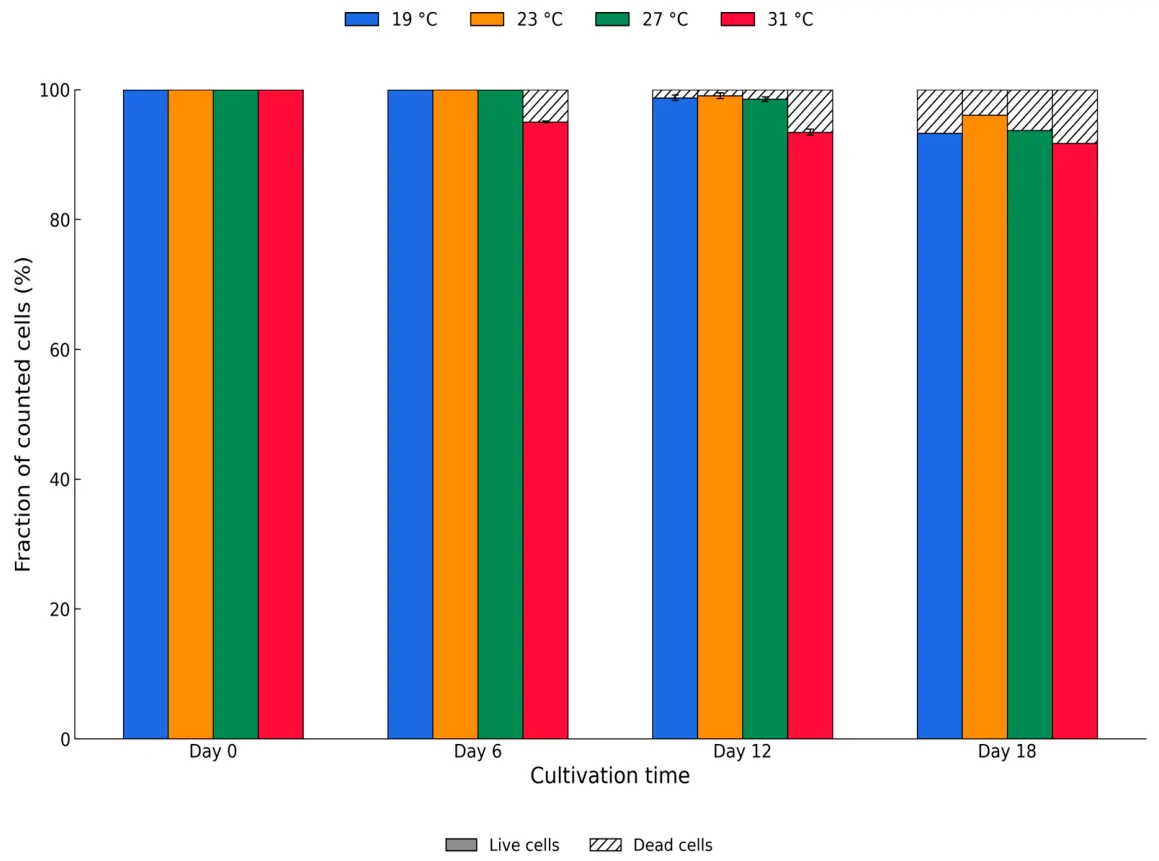
Viable and non-viable fractions of *Chlorella vulgaris* during cultivation at different temperatures. Colors identify the temperature treatments. For days 0, 6, and 12, values are means of two independent photobioreactors and error bars show the standard deviation where non-zero; day 18 represents one group-level observation per temperature and is shown without an error bar.

At the beginning of cultivation, no dead cells were detected in the analyzed samples from any temperature treatment. During subsequent cultivation, the pattern of viability loss depended on temperature (Figure 4). Live and dead cells were counted without sample dilution in five large squares of the Goryaev chamber, corresponding to a counted volume of 2 × 10^−5^ mL. The total number of cells counted per sample was 27–33 on day 0, 109–201 on day 6, and 224–353 on day 12; the day-18 group-level counts were 371, 484, 412, and 362 cells at 19, 23, 27, and 31 °C, respectively. Count-derived total cell concentrations therefore ranged from 1.35–1.65 × 10^6^ cells mL^−1^ on day 0, 5.45–10.05 × 10^6^ cells mL^−1^ on day 6, 11.20–17.65 × 10^6^ cells mL^−1^ on day 12, and 18.10–24.20 × 10^6^ cells mL^−1^ for the group-level day-18 measurements. In particular, the day-18 count-derived cell densities at 19 and 31 °C were very similar (18.55 and 18.10 × 10^6^ cells mL^−1^, respectively), whereas the corresponding OD_750_-derived biomass estimates were 5.43 and 4.05 g L^−1^. These two indirect metrics therefore cannot be quantitatively reconciled in the present dataset and are not used to validate one another or to calculate biomass per cell.

On day 6, no dead cells were detected in the counted samples at 19, 23, or 27 °C. At 31 °C, dead cells were detected in both photobioreactors, and the mean fraction of viable cells was approximately 95%.

By day 12, a small dead-cell fraction was observed in all treatments. Mean viability was approximately 98.8, 99.0, 98.5, and 93.4% at 19, 23, 27, and 31 °C, respectively. Thus, the most pronounced reduction in viability remained associated with 31 °C.

On day 18, the viable-cell fraction was 93.3% at 19 °C, 96.1% at 23 °C, 93.7% at 27 °C, and 91.7% at 31 °C. Only one group-level observation was available for each temperature treatment at this time point; therefore, these values are interpreted descriptively.

### 2.4. Relationship Between Growth and Cell Viability

The strongest reduction in biomass accumulation occurred at 31 °C and was accompanied by an earlier appearance of non-viable cells. However, biomass concentration and percentage viability represent different quantities and cannot be compared directly on a percentage basis.

Accordingly, the viability data are interpreted as descriptive evidence that loss of cell integrity appeared earlier at 31 °C. They are not used to quantify the contribution of mortality to the biomass deficit or to infer a specific sublethal physiological mechanism.

### 2.5. Culture-Medium pH Dynamics

**Figure 5:**
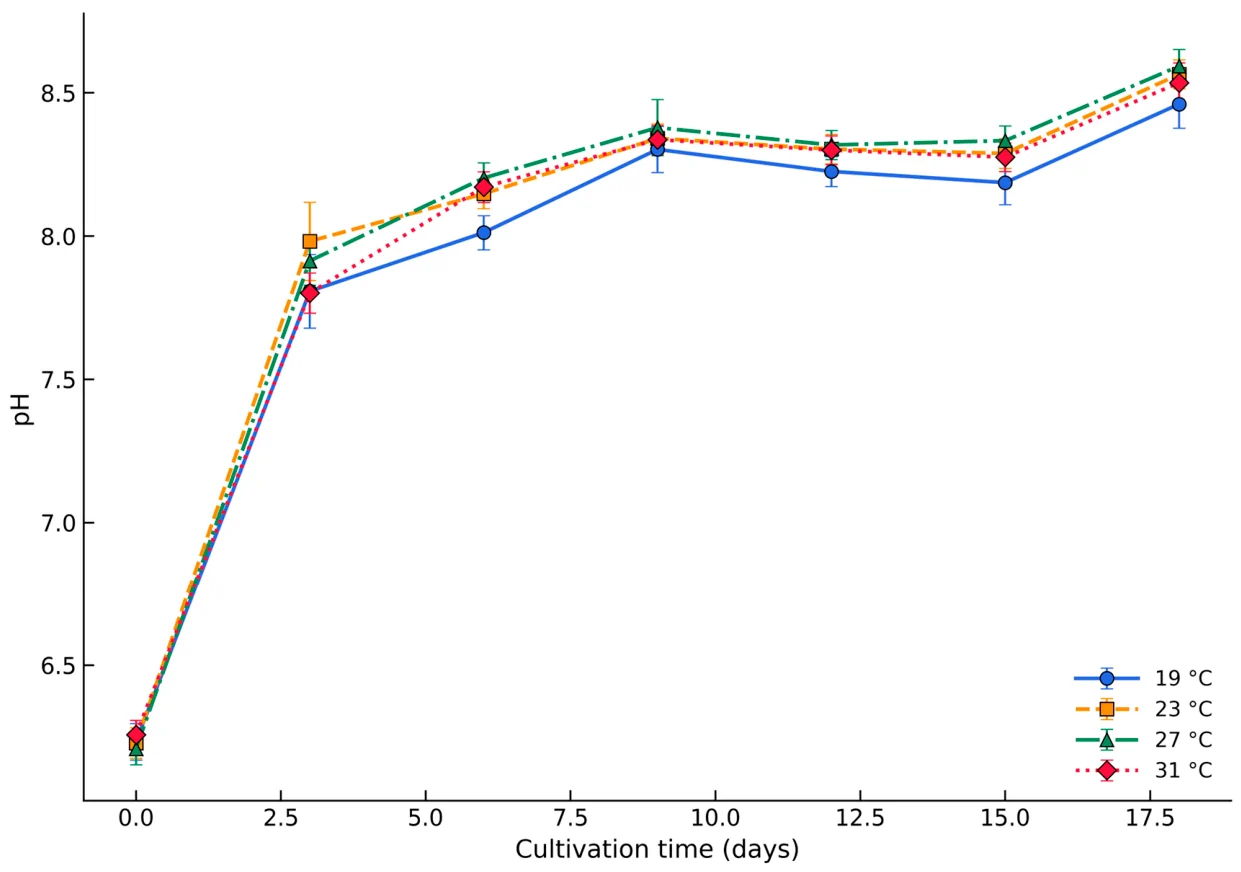
Culture-medium pH dynamics during *Chlorella vulgaris* cultivation at different temperatures. Values are means of two independent photobioreactors; error bars show the standard deviation.

All treatments showed a rapid increase in pH during the initial stage of cultivation (Figure 5). Initial values were within a narrow range of approximately 6.19–6.26. By day 3, pH had increased to approximately 7.7–8.1, after which the changes were considerably slower.

At the end of the experiment, mean pH values were approximately 8.46, 8.56, 8.59, and 8.54 at 19, 23, 27, and 31 °C, respectively. Thus, despite differences in biomass accumulation, no persistent separation of the temperature groups by final pH was observed.

The largest pH increase occurred during the first three days, after which the rate of change became comparatively small. Because this trend is directly apparent from the pH time series, the derivative pH-change figure included in the original submission was removed as redundant.

### 2.6. Chamber CO_2_ Dynamics

**Figure 6:**
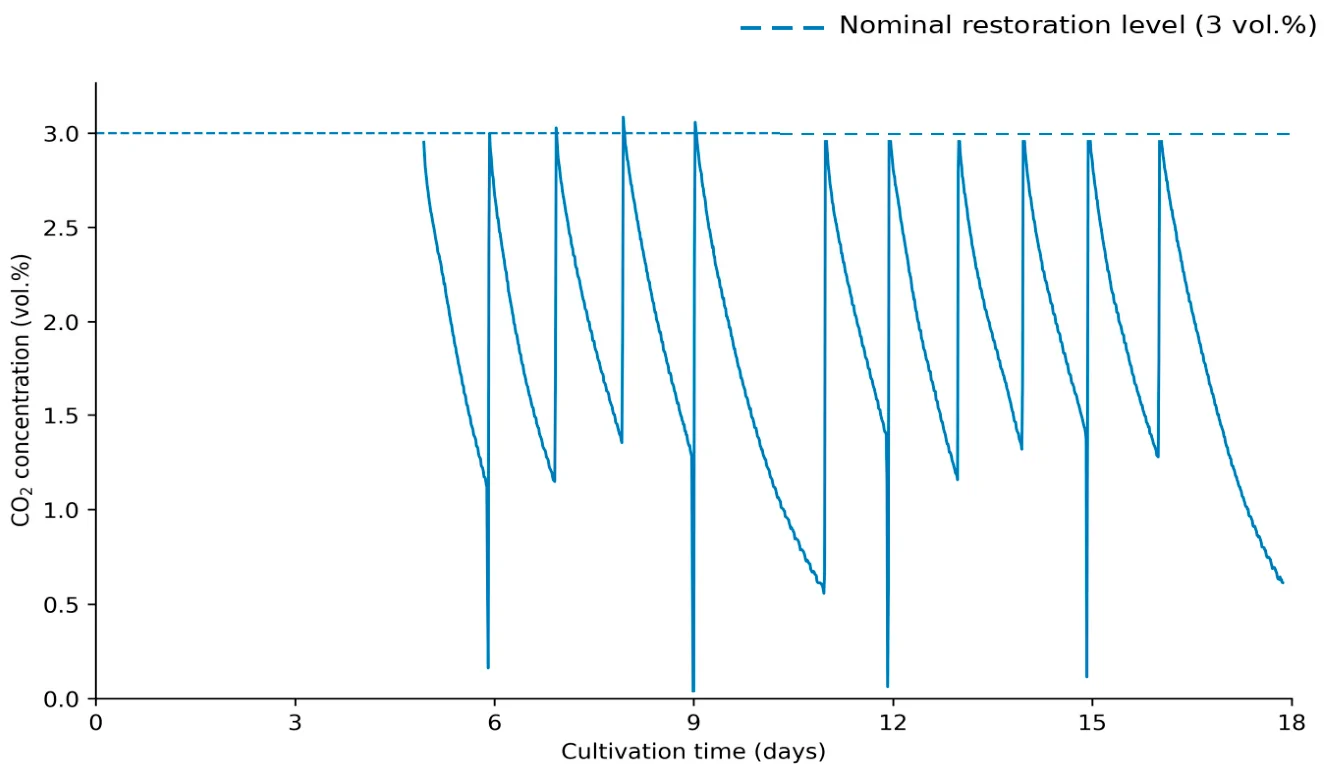
Chamber CO_2_ concentration as a function of cultivation time. The dashed horizontal line indicates the approximate restoration level (3 vol.%). The chamber was opened approximately daily for sampling associated with the present *C. vulgaris* experiment or a parallel spirulina experiment, and CO_2_ was restored after each opening. Continuous analyzer records before approximately cultivation day 5 were not retained.

Continuous chamber CO_2_ records were retained from approximately cultivation day 5 onward (Figure 6); earlier logger records were lost and were not numerically reconstructed. The chamber simultaneously contained the eight *C. vulgaris* PBRs investigated here and eight additional similarly configured PBRs with spirulina belonging to a parallel experiment. Although *C. vulgaris* was sampled every three days, the chamber was opened approximately daily for sampling associated with one or the other experiment. After each opening, the chamber was resealed and CO_2_ was restored to approximately 3 vol.%. Over the available record, mean chamber CO_2_ concentration was 1.81 ± 0.62 vol.%. The representative operating maximum was approximately 3.0 vol.%; isolated instantaneous logger spikes above this level occurred during CO_2_ replenishment and were not considered representative of the cultivation exposure. Thus, 3 vol.% represents the restoration level rather than the time-averaged exposure concentration. The subsequent decline between restoration events cannot be attributed exclusively to *C. vulgaris* uptake because the parallel spirulina cultures and chamber gas exchange could also contribute.

### 2.7. Titration-Derived Carbonate-System Alkalinity

**Figure 7:**
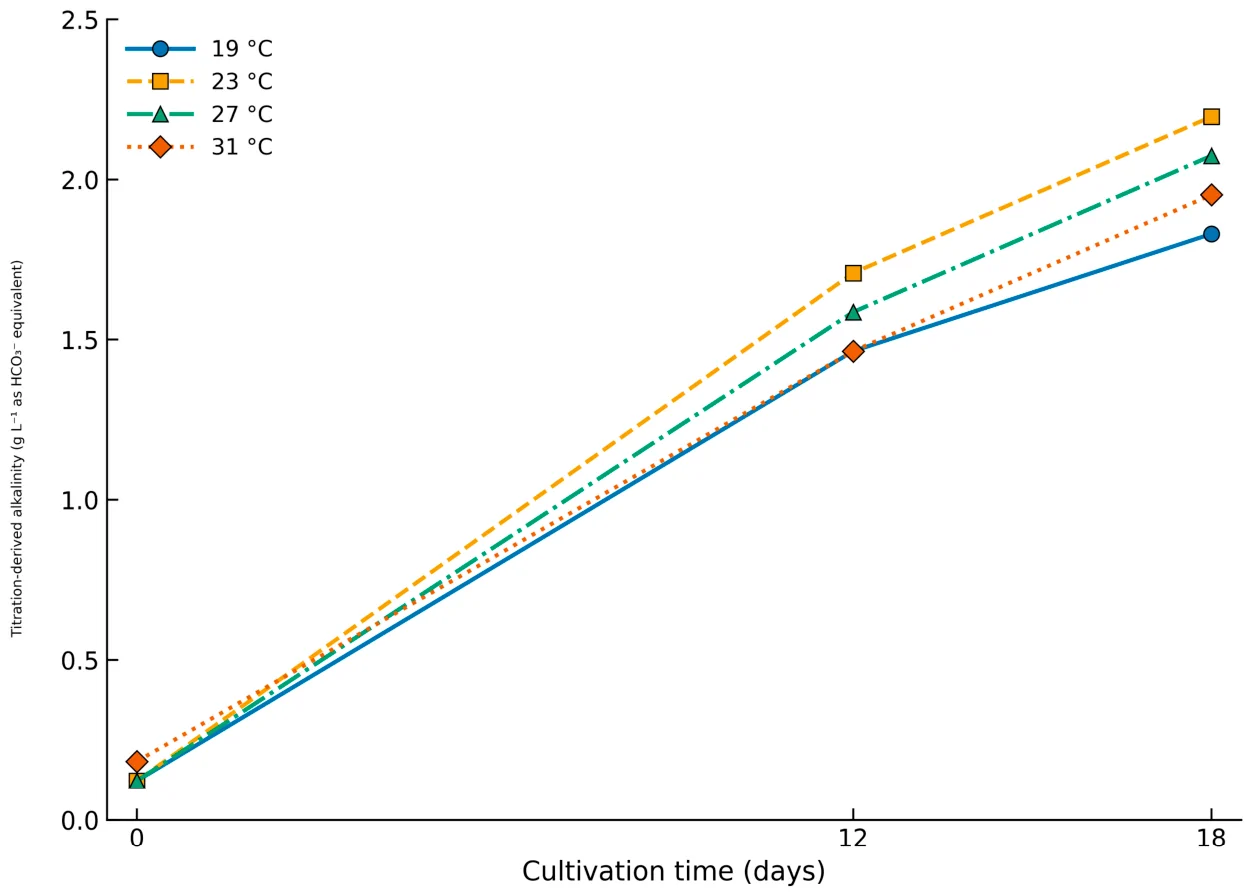
Titration-derived alkalinity of the culture medium, expressed as HCO_3_^−^ equivalent, at 19, 23, 27, and 31 °C. Values are descriptive measurements from one photobioreactor per temperature.

Acid-base titration was performed in one photobioreactor from each temperature treatment on days 0, 12, and 18. Using the carbonate-system calculation applied in the original protocol, apparent HCO_3_^−^-equivalent values increased from approximately 0.12–0.18 g L^−1^ initially to 1.46–1.71 g L^−1^ on day 12 and 1.83–2.20 g L^−1^ on day 18 (Figure 7). Because the modified Tamiya medium contains phosphate, these titration-derived values are interpreted as carbonate-system alkalinity equivalents rather than as selective direct measurements of bicarbonate.

On day 18, the apparent HCO_3_^−^-equivalent values were 1.83, 2.20, 2.07, and 1.95 g L^−1^ at 19, 23, 27, and 31 °C, respectively. No measurable phenolphthalein alkalinity was detected on days 0 and 12, whereas a small fraction nominally corresponding to approximately 0.12 g L^−1^ CO_3_^2−^ was detected on day 18.

Because these measurements were performed in only one photobioreactor per temperature and because other buffering species may contribute to the titration endpoints, they are analyzed descriptively and are not used to infer carbon sufficiency or to establish a carbon balance.

## 3. Discussion

### 3.1. Temperature Dependence of the OD_750_-Derived Growth Metric

The results demonstrate a clear temperature-dependent difference in the OD_750_-derived cultivation metric within the investigated range of 19–31 °C. Final OD_750_-derived biomass estimates were 5.43, 5.82, and 5.90 g L^−1^ at 19, 23, and 27 °C, respectively, compared with 4.05 g L^−1^ at 31 °C. With only two independent photobioreactors per treatment, the smaller differences among 19–27 °C should not be interpreted as a statistically resolved optimum. The robust observation is that the culture maintained at 31 °C produced a lower OD_750_ signal and lower OD-derived productivity; the magnitude of the corresponding dry-biomass difference is not established because the empirical conversion coefficient was not validated across temperatures.

This response is consistent with the generally nonlinear relationship between microalgal growth and temperature. Temperature-growth models typically assume a favorable temperature range followed by declining growth as the upper thermal boundary is approached [11]. Bernard and Rémond showed that at least three characteristic temperatures—minimum, optimum, and maximum—are required to describe the microalgal temperature response and that overheating can substantially reduce photobioreactor productivity [11].

A similar trend was experimentally demonstrated by Bamba et al. for *C. vulgaris* cultivated in a column photobioreactor. Specific growth rates were similar at 25 and 30 °C (0.75 and 0.76 d^−1^, respectively) but decreased to 0.63 d^−1^ at 35 °C, while volumetric productivity was reduced by more than twofold compared with 30 °C [10]. Their experiments were conducted in an 84 L column PBR with CO_2_-enriched gas, and the highest productivity at 30 °C was 0.0817 g L^−1^ d^−1^.

Recent studies further confirm that the most productive temperature range depends on the overall process configuration, including irradiance, reactor scale, gas exchange, and CO_2_ availability [14,20]. Josephine et al. observed the most intensive growth of *C. vulgaris* at 25 °C under a comparatively low light intensity of 42 µmol photons m^−2^ s^−1^, with lower biomass accumulation at higher temperatures [14]. Johnson examined temperatures from 25 to 32.5 °C and CO_2_ concentrations from 0.04 to 15% and reported a maximum productivity of 3.75 ± 0.093 g L^−1^ d^−1^ at 30 °C and 6% CO_2_; productivity declined sharply at 32.5 °C despite further increases in CO_2_ concentration [20].

The titration data provide additional descriptive context for culture-medium chemistry. Because phosphate in the Tamiya medium contributes to alkalinity, the calculated values are reported as HCO_3_^−^-equivalent carbonate-system alkalinity rather than selective bicarbonate measurements. They are therefore not used as evidence that the carbon supply was non-limiting.

For a detailed comparison with published *C. vulgaris* cultivation studies, the principal reactor and operating conditions are retained in Appendix A.2 (Table A1).

Absolute volumetric productivity differs substantially among cultivation systems because reactor scale, irradiance, aeration, CO_2_ supply, cultivation duration, and biomass-calibration methods differ simultaneously. In the present study, average OD_750_-derived productivity ranged from 0.282–0.308 g L^−1^ d^−1^ at 19–27 °C and was 0.205 g L^−1^ d^−1^ at 31 °C. These values are therefore used as within-system OD-derived metrics rather than as temperature-specific gravimetric productivity measurements or to define a universal temperature optimum.

The shape of the temperature response is more consistent across studies. Bamba et al. observed similar performance at 25 and 30 °C followed by a sharp decrease at 35 °C [10]; Josephine et al. reported maximum growth at 25 °C followed by deterioration at higher temperatures [14]; and Johnson observed a productivity decline at 32.5 °C even under elevated CO_2_ [20]. The present data likewise show a marked reduction in the OD_750_-derived metric at the upper tested temperature, while the 19–27 °C treatments remain comparatively close.

### 3.2. Temperature Alters the Temporal Structure of Culture Growth

The final OD_750_-derived biomass estimate alone does not fully describe the temperature response. An important result of this study is that differences among temperature treatments appeared during specific stages of cultivation and changed over time.

During days 0–3, interval-specific growth rates ranged from 0.374 to 0.433 d^−1^ across the four treatments. The calculated specific growth rate then decreased under all regimes; because this decline is partly inherent to the logarithmic growth-rate expression as biomass accumulates, interval volumetric productivity was additionally evaluated. This direct metric showed that the 31 °C treatment increasingly diverged after approximately day 6 and reached only 0.114 g L^−1^ d^−1^ during days 15–18, compared with 0.293–0.309 g L^−1^ d^−1^ at 19–27 °C.

Thus, the lower final OD_750_-derived estimate at 31 °C developed progressively rather than appearing only at the end of cultivation. The combined use of the coefficient-independent specific growth-rate ratio and the coefficient-dependent interval OD-derived productivity provides complementary views of this temporal divergence.

Temperature can affect photosynthetic growth, respiration, cell size, pigmentation, and other cellular properties that can influence both biomass formation and optical density [13]. Such mechanisms may contribute to the observed cultivation response. However, these functional and morphological parameters were not measured directly here; consequently, the present data do not identify which physiological process or optical property caused the lower OD_750_-derived response at 31 °C.

Absolute specific growth-rate values should nevertheless be compared cautiously across studies because they depend on strain, irradiance, CO_2_ concentration, medium composition, reactor geometry, and calculation method. For the present study, the differences among the time profiles of the four temperature treatments within the same experimental system are therefore more informative than direct comparison with published maximum growth rates.

### 3.3. Temperature Dependence of Cell Viability and Its Relation to the OD_750_ Response

In addition to differences in the OD_750_-derived growth metric, the temperature treatments differed in the temporal dynamics of cell viability. Dead cells were essentially absent from the analyzed samples at the beginning of cultivation. By day 6, however, dead cells were detected in both independent photobioreactors at 31 °C, whereas none were detected in the counted samples at 19, 23, or 27 °C. Mean viability at 31 °C at this stage was approximately 95%.

By day 12, viability had decreased in all temperature treatments, but the reduction remained most pronounced at 31 °C: approximately 93.4% compared with 98.5–99.0% at 19–27 °C. On day 18, the minimum viability was again observed at 31 °C (91.7%). The final comparison should be treated as descriptive because only one group-level measurement was available per temperature treatment at day 18.

The earlier appearance of dead cells at 31 °C is consistent with the findings of Serra-Maia et al., who reported a pronounced effect of temperature on both growth and mortality of *C. vulgaris* in a photobioreactor [12]. In that study, the viable-cell fraction decreased as temperature increased, and growth of the viable population and cell mortality were analyzed separately.

The absolute reduction in viability in the present study was nevertheless smaller than that reported by Serra-Maia et al. This difference does not necessarily represent a contradiction because thermal sensitivity depends on strain, illumination, medium composition, photobioreactor design, and the viability-assessment method. Serra-Maia et al. used fluorescein diacetate staining and also reported temperature-related physiological changes in the viable fraction of the population [12].

The viability observations therefore provide a qualitative temporal indication of increased cell damage at 31 °C, but they should not be quantitatively compared with the percentage difference in the OD_750_-derived biomass estimate because the two variables have different normalization bases.

Likewise, the viability data alone cannot distinguish increased mortality from changes in photosynthesis, respiration, or cell-division rate. Measurements such as chlorophyll fluorescence, oxygen exchange, or direct division-rate analysis would be required for a mechanistic interpretation.

Accordingly, the interpretation is limited to the directly supported observations that non-viable cells appeared earlier at 31 °C and that the OD_750_-derived growth metric was lower at this temperature. The discrepancy between absolute cell counts and the OD-derived estimate is particularly important at day 18: count-derived cell densities were 18.55 × 10^6^ cells mL^−1^ at 19 °C and 18.10 × 10^6^ cells mL^−1^ at 31 °C, despite OD_750_-derived biomass estimates of 5.43 and 4.05 g L^−1^, respectively. Thus, the data do not support interpreting the OD difference as a proportional difference in cell abundance. Without contemporaneous dry-weight calibration, cell-size measurements, or an independently validated absolute counting method, possible contributions from dry mass per cell, cell size, pigmentation/scattering properties, aggregation, or counting uncertainty cannot be separated. The main temperature effect is therefore reported as a lower OD_750_-derived response at 31 °C rather than as a validated dry-biomass deficit.

### 3.4. Role of Elevated CO_2_ and Culture-Medium Chemistry

The temperature response in this experiment developed under an elevated but dynamically varying CO_2_ gas-phase regime. The chamber was opened approximately daily because it simultaneously supported the present *C. vulgaris* experiment and a parallel spirulina experiment. CO_2_ was restored to approximately 3 vol.% after each opening, producing the repeated restoration-and-decline pattern shown in Figure 6. For the retained record from approximately day 5 onward, the time-averaged concentration was 1.81 ± 0.62 vol.%. The observed temperature response therefore applies to this intermittently restored elevated-CO_2_ regime rather than to constant exposure at 3 vol.%.

CO_2_ concentration can substantially alter the growth of *C. vulgaris*, and the temperature response may depend on inorganic-carbon supply [10,15,16]. Bamba et al. showed that supplemental CO_2_ supported comparatively high productivity up to 30 °C, while further warming to 35 °C reduced growth [10]. Recent datasets covering multiple combinations of temperature and CO_2_ concentration also demonstrate that biomass productivity depends strongly on both variables [20]. Our earlier chamber study additionally showed that *C. vulgaris* can be cultivated over a broad gas-phase CO_2_ range from 0.04 to 9 vol.% in the same 12 m^3^ chamber configuration [19].

Additional context is provided by our two previous *C. vulgaris* studies conducted under atmospheric CO_2_. The detailed inter-experiment comparison has been moved from the main Discussion to Appendix A.3 (Table A2), while the principal implications are retained below.

In the aeration study conducted under atmospheric CO_2_ (~0.04%), the most productive regimes provided an approximate average net volumetric productivity of 0.14–0.16 g L^−1^ d^−1^, with biomass concentrations of about 4.5–5.0 g L^−1^ reached at aeration intensities of approximately 0.9–2.0 vvm [7]. In the subsequent initial-biomass study, also performed under atmospheric CO_2_ and at a fixed aeration rate of 0.5 vvm, the approximate average productivity was 0.065–0.113 g L^−1^ d^−1^ [8].

In the present experiment, average OD_750_-derived net volumetric productivity was 0.282, 0.303, and 0.308 g L^−1^ d^−1^ at 19, 23, and 27 °C, respectively, despite a considerably lower nominal bubbling intensity of 0.10 vvm. This inter-experiment comparison is descriptive because the previous studies and the present experiment differ not only in CO_2_ regime and reactor configuration but also in biomass-calibration context. Accordingly, the higher numerical OD-derived productivity values observed here cannot be assigned quantitatively to the change in CO_2_ availability alone.

However, this comparison is not a controlled factorial experiment on the effect of CO_2_. The experimental series differed simultaneously in working volume and reactor geometry, temperature regime, irradiance, initial biomass concentration, cultivation duration, and gas-supply configuration. Therefore, the higher productivity observed in the present study cannot be quantitatively attributed to the change from atmospheric ~0.04% CO_2_ to the restoration of the chamber atmosphere to 3 vol.% CO_2_. The differences should be viewed as an inter-experiment trend rather than an estimate of an independent CO_2_ effect.

The direct CO_2_ record confirms that 3 vol.% represented a restoration level rather than a constant exposure concentration. The comparatively rapid declines between some replenishment events cannot be assigned to the eight *C. vulgaris* cultures alone because eight additional spirulina PBRs shared the chamber and gas exchange from the chamber may also have contributed. Because the logger records from approximately the first five cultivation days were not retained, no numerical reconstruction of the missing early CO_2_ time series was attempted. Dissolved inorganic carbon and mass-transfer rates were not measured continuously, so the experiment does not quantitatively separate temperature effects from possible temperature-dependent differences in carbon transfer and utilization.

The pH dynamics further indicate that culture-medium chemistry changed substantially during the initial stage of cultivation. Under all temperature regimes, pH rapidly increased from approximately 6.2 to 7.7–8.1 during the first three days and subsequently remained mainly within 8.0–8.6. No pronounced separation among temperature treatments was observed by the end of the experiment.

This pattern also differs from our previous experiments under atmospheric CO_2_, in which final pH increased to approximately 9.5–9.9 [7,8]. In the present study, final values were approximately 8.5–8.6. The lower pH is qualitatively consistent with greater CO_2_ availability because CO_2_ supply counteracts strong alkalization of the culture medium.

Overall, comparison with our previous experiments and with published studies indicates that elevated inorganic-carbon availability can influence culture performance but does not eliminate temperature sensitivity. In the present system, the clearest supported effect was the reduced OD_750_-derived response at 31 °C.

## 4. Materials and Methods

### 4.1. Microalgal Culture and Growth Medium

A culture of *Chlorella vulgaris* obtained from the microalgal culture collection of the Faculty of Geography, Lomonosov Moscow State University, was used. No additional strain designation was available for this culture. *C. vulgaris* is a unicellular green microalga with predominantly isometric or slightly ellipsoidal cells approximately 2–7 µm in diameter.

A modified Tamiya medium was used for cultivation. Per liter of distilled water, the medium contained 5.0 g KNO_3_, 1.25 g KH_2_PO_4_, 2.5 g MgSO_4_·7H_2_O, 0.009 g FeSO_4_·7H_2_O, 0.037 g EDTA, 2.86 mg H_3_BO_3_, 1.81 mg MnCl_2_·4H_2_O, 0.22 mg ZnSO_4_·7H_2_O, 0.018 mg (NH_4_)_6_Mo_7_O_24_·4H_2_O, and 0.023 mg NH_4_VO_3_. The initial medium pH was approximately 6.

Before the experiment, the culture was maintained under laboratory conditions at approximately 25 °C with atmospheric air and used to prepare the inoculum. No temperature-specific pre-acclimation to the four experimental regimes was performed. All photobioreactors were inoculated simultaneously to provide comparable initial biomass concentrations across treatments.

### 4.2. Experimental Conditions

**Figure 8:**
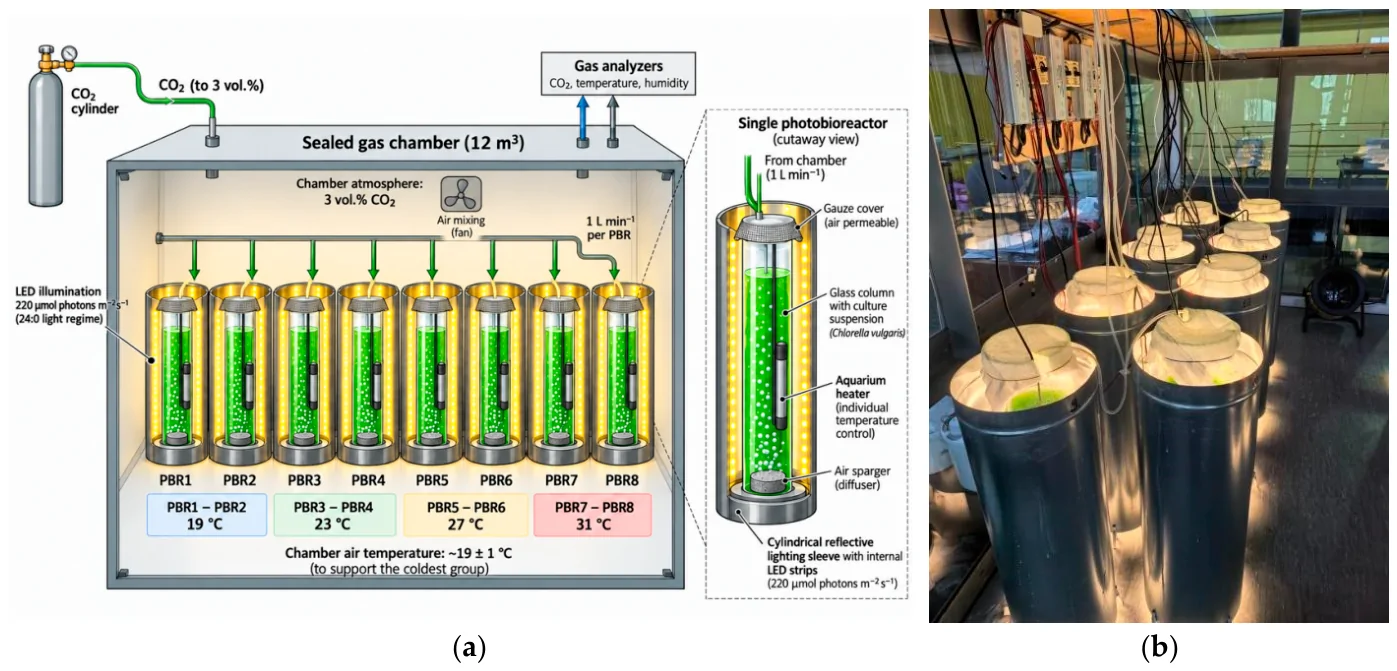
Experimental setup used for *Chlorella vulgaris* cultivation under elevated CO_2_: (**a**) AI-assisted schematic illustration and subsequently checked by the authors against the actual experimental configuration; (**b**) photograph of the actual photobioreactor array inside the chamber.

The experiment was conducted in a sealed 12 m^3^ gas chamber equipped with sensors for temperature, relative humidity, and pressure and with gas analyzers monitoring O_2_, CO, CO_2_, NH_3_, CH_4_, SO_2_, and NO_2_. The chamber was connected to CO_2_ cylinders used to establish an elevated-CO_2_ atmosphere (Figure 8). During the present experiment, the chamber simultaneously housed the eight *C. vulgaris* photobioreactors described here and eight additional similarly configured photobioreactors containing spirulina as part of a separate parallel experiment.

Eight cylindrical glass column photobioreactors obtained from InnTech (Reutov, Russia) with a working volume of 10 L and a height of 80 cm were placed inside the chamber. The upper part of each reactor was covered with a 20 × 20 cm gauze cover. An air sparger installed at the bottom provided continuous bubbling with gas drawn directly from the chamber atmosphere. The gas flow rate was 1 L min^−1^ per photobioreactor (0.10 vvm).

Illumination was supplied continuously (24:0 light:dark) by LED sources mounted inside reflective cylindrical sleeves surrounding the photobioreactors. Photosynthetically active radiation (PAR) was measured individually at all eight *C. vulgaris* PBR positions before the experiment. At the reporting precision retained for the experiment, all positions were approximately 220 µmol photons m^−2^ s^−1^, with no practically relevant position-specific difference detected.

### 4.3. Experimental Design

The cultivation period was 18 days. Four nominal temperature regimes were investigated: 19, 23, 27, and 31 °C. Each treatment was represented by two independent photobioreactors: PBR1–PBR2 at 19 °C, PBR3–PBR4 at 23 °C, PBR5–PBR6 at 27 °C, and PBR7–PBR8 at 31 °C. The temperature treatments occupied fixed PBR positions and were not randomized.

**Figure A1:**
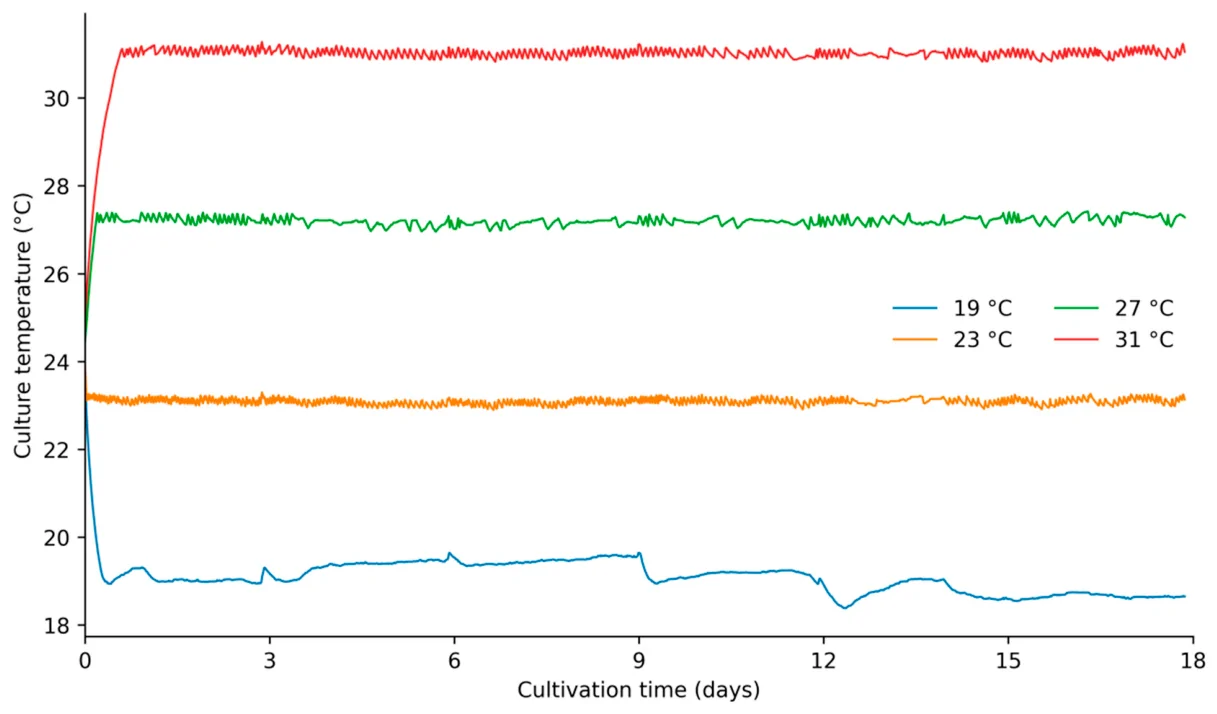
Continuous recorded culture temperatures for the four temperature groups as a function of cultivation time.

The culture temperature was regulated using immersed aquarium heaters. One continuous temperature sensor monitored each temperature pair. Over the experiment, recorded mean ± SD temperatures were 19.12 ± 0.42, 23.09 ± 0.09, 27.19 ± 0.20, and 30.96 ± 0.44 °C for the nominal 19, 23, 27, and 31 °C groups, respectively. The complete time series is provided in Appendix A.1 (Figure A1).

Before cultivation, the chamber gas-phase CO_2_ concentration was adjusted to approximately 3 vol.%. The *C. vulgaris* reactors were sampled every three days at approximately the same time of day; however, because samples were also collected from the parallel spirulina experiment, the chamber was opened approximately daily. After each chamber-opening event, the chamber was resealed and CO_2_ was supplied until the concentration was restored to approximately 3 vol.%. CO_2_ was not controlled at a constant set point between openings and therefore declined between restoration events. Continuous CO_2_ logger records were retained only from approximately cultivation day 5 onward because the earlier digital records were lost. Over the retained record, mean CO_2_ concentration was 1.81 ± 0.62 vol.%. The representative operating maximum was approximately 3.0 vol.%; brief instantaneous logger values above this level during replenishment were not treated as representative experimental exposure. The same chamber-opening and CO_2_-restoration procedure was used throughout the experiment, but no numerical reconstruction of the missing first five days was performed.

### 4.4. Biomass Concentration, pH, and Temperature Measurements

Optical density at 750 nm (OD_750_) was measured using an SF-102 spectrophotometer obtained from Akvilon (Podol’sk, Russia) on days 0, 3, 6, 9, 12, 15, and 18. When necessary, samples were diluted with distilled water to maintain OD within the linear measurement range of approximately 0.3–0.6.

Biomass concentration was calculated using the empirical calibration relationship:

X = 0.4 · OD_750_ · n (1)

where X is the OD_750_-derived biomass estimate (g L^−1^), 0.4 is an empirical calibration coefficient previously established in our laboratory for the *C. vulgaris* culture used in this experimental series from paired OD_750_ and gravimetric dry-weight measurements and subsequently applied in our previous cultivation studies [7,8], and n is the sample dilution factor. The historical calibration comprised 10 concentration points covering approximately 0.1–4.0 g L^−1^ dry biomass, with two replicate measurements at each concentration point (20 paired OD_750_/dry-weight measurements in total); linear regression gave R^2^ = 0.982. Thus, the coefficient is based on an experimental dry-weight calibration rather than a literature-assumed value. However, no parallel gravimetric dry-weight measurements were performed in the present experiment, and the coefficient was not independently revalidated at 19, 23, 27, and 31 °C. Accordingly, the reported g L^−1^ values are treated as OD_750_-derived estimates rather than temperature-specific gravimetric biomass measurements. The discrepancy between these estimates and the count-derived cell concentrations is reported explicitly in the Results and Discussion.

Culture-medium pH was measured with a pH-150MI pH meter (obtained from Izmteh, Moscow, Russia, accuracy ±0.05) at the same sampling points as OD_750_. Temperature was recorded continuously for each temperature pair as described above. All sampling and analytical measurements were performed according to the same protocol for every reactor.

### 4.5. Microscopic Analysis and Cell Viability

Cell viability was evaluated on days 0, 6, 12, and 18 by microscopic counting of live and dead cells. Microscopy was performed using a Levenhuk MED Series light microscope (Levenhuk, Tampa, FL, USA) at ×100 and ×400 magnification.

Cell viability was assessed by differential methylene-blue staining based on differences in membrane permeability between viable and non-viable cells. Cells were counted using a hemocytometer obtained from Minimed (Bryansk region, Russia, depth 0.1 mm; large-square side length 0.2 mm). No sample dilution was applied. For each analyzed sample, live and dead cells were counted in five large squares, corresponding to a total counted volume of 2 × 10^−5^ mL. Count-derived cell concentration was calculated as N × 50,000 cells mL^−1^, where N is the total number of cells counted in the five squares. Microscopic counting was performed primarily to determine the live/dead fraction and was not independently validated as an absolute cell-density method.

The viable-cell fraction was calculated as follows:

(2) $$V=\frac{N_{\mathrm{live}}}{N_{\mathrm{live}}+N_{\mathrm{dead}}}\times 100\%$$

where *N*_live_ and *N*_dead_ are the numbers of live and dead cells, respectively. On days 0, 6, and 12, counts were performed for individual photobioreactors. On day 18, one group-level observation was available for each temperature treatment; these values were therefore used descriptively.

### 4.6. Growth Parameters and Data Processing

The interval-specific growth rate and pH change rate were first calculated separately for each photobioreactor over consecutive sampling intervals:

(3) $$\mu_{i}=\frac{\ln\left(X_{i+1}/X_{i}\right)}{t_{i+1}-t_{i}}$$

(4) $${\left(\frac{\mathrm{dpH}}{\mathrm{dt}}\right)}_{i}=\frac{pH_{i+1}-pH_{i}}{t_{i+1}-t_{i}}$$

where X_i_ and X_i+1_ are successive OD_750_-derived biomass estimates and t_i_ and t_i+1_ are the corresponding cultivation times. Specific growth rate was expressed in d^−1^. Because the same multiplicative OD_750_-to-biomass coefficient is present in both X terms, it cancels from the logarithmic specific-growth-rate expression. Interval volumetric productivity was additionally calculated as P_i_ = (X_i+1_ − X_i_)/(t_i+1_ − t_i_) and expressed in g L^−1^ d^−1^; unlike μ, this absolute OD-derived productivity scales directly with the empirical conversion coefficient.

Average net volumetric biomass productivity over the full cultivation period was calculated as follows:

(5) $$P_{\mathrm{avg}}=\frac{X_{f}-X_{0}}{t_{f}-t_{0}}$$

where X_0_ and Xf are the initial and final biomass concentrations (g L^−1^), respectively, and t is the cultivation duration (d). Productivity was expressed in g L^−1^ d^−1^. Results were calculated separately for each photobioreactor and then summarized as the mean and standard deviation of the two independent reactors within each temperature treatment. Average net volumetric productivity calculated independently for each PBR was compared among temperature treatments using one-way ANOVA followed by Tukey’s HSD test. Because only two independent PBRs were available per treatment, inferential results were interpreted cautiously and together with the individual reactor values; no resolved optimum within 19–27 °C is claimed.

### 4.7. Titration-Derived Carbonate-System Alkalinity

Carbonate-system alkalinity was assessed by sequential acid-base titration with 0.1 N HCl using 5 mL culture samples. Measurements were performed on days 0, 12, and 18 for one photobioreactor from each temperature treatment (PBR1, PBR3, PBR5, and PBR7). The original carbonate/bicarbonate calculation was retained to express apparent CO_3_^2−^ and HCO_3_^−^ equivalents, but because the modified Tamiya medium contains phosphate, these values are interpreted as titration-derived alkalinity equivalents rather than selective direct measurements of carbonate species.

Each sample was first titrated to the phenolphthalein endpoint, corresponding to conversion of carbonate ions to bicarbonate, and the endpoint was identified by disappearance of the phenolphthalein color. Titration was then continued to the methyl-orange endpoint, corresponding to further neutralization of bicarbonate to carbonic acid.

For samples in which phenolphthalein alkalinity was less than one-half of total alkalinity, carbonate and bicarbonate concentrations were calculated as follows:

(6) $$C_{CO_{3}^{2-}}=\frac{60000NV_{P}}{V_{s}}$$

(7) $$C_{\mathrm{HC}O_{3}^{-}}=\frac{61000N\left(V_{T}-2V_{P}\right)}{V_{s}}$$

where N is the HCl normality, *V_P_* is the acid volume consumed to the phenolphthalein endpoint (mL), *V_T_* is the total acid volume consumed to the methyl-orange endpoint (mL), and *V_s_* is the analyzed sample volume (mL). Concentrations were expressed in mg L^−1^.

## 5. Conclusions

Under the investigated elevated and intermittently restored CO_2_ regime, *Chlorella vulgaris* showed broadly comparable OD_750_-derived growth metrics at 19–27 °C, whereas cultivation at 31 °C consistently produced a lower OD_750_ signal and lower OD-derived productivity. Non-viable cells appeared earlier at 31 °C, but the descriptive viability measurements do not identify the physiological mechanism responsible for this response. Importantly, although the empirical OD_750_-to-biomass coefficient had previously been established from gravimetric dry-weight calibration for this culture, it was not revalidated separately at each temperature in the present experiment, and the day-18 count-derived cell densities at 19 and 31 °C were nearly identical despite different OD-derived biomass estimates. The magnitude of the 31 °C effect therefore cannot be interpreted as a validated dry-biomass deficit. The results support a temperature-dependent OD_750_/cultivation response at 31 °C within this photobioreactor system, while the contributions of dry mass per cell, cell size, optical properties, and other physiological changes remain unresolved.

## Data Availability

The underlying per-reactor OD_750_-derived biomass estimates, pH, growth rate, productivity, and raw live/dead cell counts for each available reactor and sampling day, together with the recorded culture-temperature and chamber-CO_2_ logs used in this study, are provided as Supporting Data S1 accompanying the revised manuscript.

## Appendix A

### Appendix A.1. Logged Culture Temperatures

Continuous temperature records for the four treatment groups are shown in Figure A1. Each trace represents the sensor assigned to one pair of photobioreactors. Short excursions at the beginning of cultivation and during chamber servicing are visible in the raw record; the mean temperatures over the full experiment were 19.12 ± 0.42, 23.09 ± 0.09, 27.19 ± 0.20, and 30.96 ± 0.44 °C for the nominal 19, 23, 27, and 31 °C treatments, respectively.

### Appendix A.2. Comparison with Published Cultivation Studies

**Table A1:** Comparison of the present study with published experimental studies on *Chlorella vulgaris* cultivation.

Study	Cultivation System/Working Volume	Temperature	CO_2_ Regime	Aeration	Light Intensity	Photoperiod	Cultivation Time	Maximum/Final Biomass	Average Biomass Productivity	Main Observation
Present study	Column glass PBR/10 L	19, 23, 27, 31 °C	Restored to ~3% CO_2_; mean 1.81%	0.10 vvm	220 µmol photons m^−2^ s^−1^	Continuous	18 d	OD_750_-derived: 5.43, 5.82, 5.90, 4.05 g L^−1^	OD_750_-derived: 0.282, 0.303, 0.308, 0.205 g L^−1^ d^−1^	19–27 °C similar; lower OD_750_ metric at 31 °C
Pavlov et al., 2025 [7]	Cylindrical glass PBR/4 L	24.5–28.5 °C	Atmospheric air (~0.04% CO_2_)	0.25–3.25 vvm	~300 µmol photons m^−2^ s^−1^	Continuous	30 d	~4.5–5.0 g L^−1^ at ~0.9–2.0 vvm	~0.14–0.16 g L^−1^ d^−1^ *	High aeration allowed high biomass accumulation under atmospheric CO_2_
Pavlov et al., 2026 [8]	Cylindrical glass PBR/4 L	~22 °C	Atmospheric air (~0.04% CO_2_)	0.50 vvm	~300 µmol photons m^−2^ s^−1^	Continuous	15 d	~1.5–1.7 g L^−1^	~0.065–0.113 g L^−1^ d^−1^ *	Biomass productivity remained lower under atmospheric CO_2_ and fixed aeration
Bamba et al., 2015 [10]	Column PBR/84 L	25, 30, 35 °C	CO_2_-enriched gas	~200 L h^−1^ in temperature experiment	336 µmol photons m^−2^ s^−1^	Continuous	~8–12 d to maximum biomass	~0.95, 1.00, 0.45 g L^−1^	0.0705, 0.0817, 0.0358 g L^−1^ d^−1^	Similar growth at 25–30 °C; marked inhibition at 35 °C
Josephine et al., 2022 [14]	Polycarbonate carboy/20 L	20, 25, 30, 35, 40 °C	Atmospheric air (~0.04% CO_2_)	Not reported	42 µmol photons m^−2^ s^−1^	Artificial fluorescent illumination	15 d	~1.0 g L^−1^ by day 10 at 25 °C	~0.10 g L^−1^ d^−1^ †	Highest growth at 25 °C; reduced biomass at ≥35 °C
Johnson, 2026 [20]	Bubble culture/300 mL	25, 27.5, 30, 32.5 °C	0.04–15% CO_2_	Gas aeration	300 µmol photons m^−2^ s^−1^	Continuous	Factorial dataset	Not reported	Maximum 3.75 ± 0.093 g L^−1^ d^−1^ at 30 °C and 6% CO_2_	Strong CO_2_ dependence; productivity declined sharply at 32.5 °C

* Approximate values derived from reported initial/final biomass and cultivation time. † Approximate gross rate based on the reported biomass trajectory.

### Appendix A.3. Comparison with Previous Experiments

**Table A2:** Comparison of the present study with our previous *Chlorella vulgaris* experiments.

Parameter	Pavlov et al., 2025 [7]	Pavlov et al., 2026 [8]	Present Study
Main investigated factor	Aeration intensity	Initial biomass concentration	Cultivation temperature
Working volume	4 L	4 L	10 L
Cultivation temperature	24.5–28.5 °C	~22 °C	19, 23, 27, and 31 °C
CO_2_ regime	Atmospheric air (~0.04% CO_2_), no enrichment	Atmospheric air (~0.04% CO_2_), no enrichment	Elevated chamber atmosphere intermittently restored to ~3 vol.% CO_2_ after chamber openings; recorded mean 1.81 vol.% from day ~5 onward
Aeration intensity	0.25–3.25 vvm	0.50 vvm	1 L min^−1^ = 0.10 vvm
Light intensity	300 µmol photons m^−2^ s^−1^	300 µmol photons m^−2^ s^−1^	220 µmol photons m^−2^ s^−1^
Photoperiod	Continuous	Continuous	Continuous
Cultivation duration	30 d	15 d	18 d
Initial biomass concentration	0.33 g L^−1^	0.012, 0.053, 0.110, and 0.530 g L^−1^	0.36 g L^−1^
Highest observed biomass concentration	4.5–5.0 g L^−1^ at 0.9–2.0 vvm	1.5–1.7 g L^−1^	Highest OD_750_-derived biomass estimate: 5.90 g L^−1^ at 27 °C
Average net biomass productivity	0.14–0.16 g L^−1^ d^−1^ *	0.065–0.113 g L^−1^ d^−1^ *	OD_750_-derived: 0.282, 0.303, 0.308, and 0.205 g L^−1^ d^−1^ at 19, 23, 27, and 31 °C
pH evolution	6.5 → ~9.5	5.3–6.9 → 9.5–9.9	6.2 → ~8.5–8.6
Main outcome	Biomass accumulation increased with aeration and approached a plateau at ~1.3 vvm	Initial biomass concentration altered growth kinetics and specific growth rate	19–27 °C broadly comparable; lower OD_750_-derived response and productivity at 31 °C; dry-biomass magnitude not independently validated

* Approximate average net productivity derived from published biomass data.
